# Unsteady mix convectional stagnation point flow of nanofluid over a movable electro-magnetohydrodynamics Riga plate numerical approach

**DOI:** 10.1038/s41598-023-37575-2

**Published:** 2023-07-06

**Authors:** Saleem Nasir, Abdallah S. Berrouk, Taza Gul, Islam Zari, Wajdi Alghamdi, Ishtiaq Ali

**Affiliations:** 1 Mechanical Engineering Department, Khalifa University of Science and Technology, P.O. Box 127788, Abu Dhabi, United Arab Emirates; 2 Center for Catalysis and Separation (CeCas), Khalifa University of Science and Technology, P.O. Box 127788, Abu Dhabi, United Arab Emirates; 3 Department of Mathematics, City University of Science and Information Technology, Peshawar, 25000 Pakistan; 4 Department of Mathematics, University of Peshawar, Peshawar, 25000 Khyber Pakhtunkhwa Pakistan; 5 Department of Information Technology, Faculty of Computing and Information Technology, King Abdulaziz University, Jeddah, 80261 Saudi Arabia; 6 Department of Mathematics and Statistics College of Science, King Faisal University, P. O. Box 400, Al-Ahsa, 31982 Saudi Arabia

**Keywords:** Materials science, Mathematics and computing, Physics

## Abstract

The flow at a time-independent separable stagnation point on a Riga plate under thermal radiation and electro-magnetohydrodynamic settings is examined in this research. Two distinct base fluids-H_2_O and C_2_H_6_O_2_ and TiO_2_ nanostructures develop the nanocomposites. The flow problem incorporates the equations of motion and energy along with a unique model for viscosity and thermal conductivity. Similarity components are then used to reduce these model problem calculations. The Runge Kutta (RK-4) function yields the simulation result, which is displayed in graphical and tabular form. For both involved base fluid theories, the nanofluids flow and thermal profiles relating to the relevant aspects are computed and analyzed. According to the findings of this research, the C_2_H_6_O_2_ model heat exchange rate is significantly higher than the H_2_O model. As the volume percentage of nanoparticles rises, the velocity field degrades while the temperature distribution improves. Moreover, for greater acceleration parameters, TiO_2_/ C_2_H_6_O_2_has the highest thermal coefficient whereas TiO_2_/ H_2_O has the highest skin friction coefficient. The key observation is that C_2_H_6_O_2_ base nanofluid has a little higher performance than H_2_O nanofluid.

## Introduction

Growing amounts of investigation are being applied to improve the heat transmission performance of mechanical devices in various technical and engineering fields. To enhance the heat transmission performance of a mechanical system, scientists and engineers have offered a range of direct or indirect strategies. Several development environments, such as electrostatic blood supply^1^ and convectional transport^2,3^, require the formulation of appropriate mathematical models to assess different fluid flows corresponding to various dynamic conditions. One of the most well-known approaches to guarantee excellent thermal efficiency at a cheap cost in recent years is the use of nanofluid as a coolant in thermal systems. Conventional fluids and incredibly tiny nanometer molecules merge consistently to form nanofluid. To create the nanofluid dispersion, metals like alumina, copper, silver, titania, and others are typically used as nanoparticles in the conventional fluid water, oils. The works published by Das et al.^4^ and Yu and Xie^5^ give details on the characteristics of nanofluids. In present analysis we studied the two different based fluids H_2_O (water) as well as C_2_H_6_O_2_ (ethylene glycol) with TiO_2_ nanoscale. Blended nanocomposite is used in a spectrum of uses, particularly thermal transmission applications like photovoltaic panels, heat pipes, cooling systems, and many others^6–10^ for its highest heat transfer capabilities. But to successfully harness its unique qualities, nanofluids technology must improve. In this compliance, numerous experimental investigations on nanofluids have been conducted and reported by various groups, including Suresh et al.^11^, Gorla and Sidawi^12^, Gul et al.^13^ and Alnahdi et al.^14,15^. Using analytical, computational, and statistical methods, the mechanics of several nanofluids with distinct flow arrangement were also actively investigated. The blended copper-alumina/water, titania-alumina/water, and copper-titania/water nanofluids subjected to an exponential form velocity stretching sheet were numerically and statistically studied by Hussain et al.^16^. Bhatti et al.^17^ also investigated the composite diamond-silica/water nanofluid with an approach toward solar collector implementations. Tripathi et al.^18^ and Zeeshan et al.^19^ explored another analysis of composite nanofluid flow with microfluidic channels and lubricating oil utilization. Besides this, Hussain et al.^20–26^, Nasir et al.^27,28^ have recently published work on nanofluids boundary layer flows in several configurations.

The revolutionary phenomena known as stagnation point flow configuration is frequently seen when substance is hit to a solid surface perpendicularly or diagonally. Diverse kinds of utilization in scientific and technical concepts, such as aircraft wings and oscillatory processes, have been supported by the stagnation point hypothesis. Additionally, the designing of automotive and numerous manufacturing operations also emphasized the significance of stagnation point streams. To correctly assess the implementations, it is crucial to understand the mathematical foundation and modeling of such phenomena. The influence of fluid motion vs various shapes is reported in many studies in the literature^29–32^. Scientists appreciate steady flow in manufacturing procedures although it renders the processes easier to handle. However, real-world experience reveals that unfavorable unsteady consequences can still happen close to a gadget even in the ideal scenario of fluid flow. These unfavorable outcomes may be brought on by self-inflicted physical movements or by fluid imbalances. The two-dimensional unsteady separable stagnation point unsteady stagnation flow regarding the non-porous medium was first proposed by Ma and Hui^33^. In addition, Berrouk et al.^34^ and Zainal et al.^35^ performed multiple computational analyses of unsteady stagnation flow on the conventional and nanofluids.

Among the most recent advancements for addressing poor fluid conductance was the Riga plate; for further information, see Gailitis and Lielausus^36^. This gadget uses magnets and alternated sets of electrodes as an electromagnetic actuator. To control liquid motion, it is utilized to create an electromotive force which originates in the Lorentz force. Additionally, the Riga plate can be utilized to reduce surface friction and stop the development of instability^37^. According to a survey of the literature, Perez et al.^38^ have already experimented with the flow of nanofluid across a Riga plate. Using a Riga plate, Supian et al.^39^ investigate the electromagnetic slipping viscous flow of nanofluid. Researchers have also explored nanofluids to examine its features when the flow is structured over a Riga plate due to the problems regarding the progression of heat transmission. The radiative Hiemenz flow of copper-alumina/water nanofluid toward an EMHD Riga plate was examined by Bilal et al.^40^. The respective studies have contributed to some of the most recent research: Ragupathi et al.^41–43^ and Hakeem et al.^44,45^.

Following a discussion of the uses of stagnation point flows, Riga plates and nanofluids, a description on contemporary investigation examines the effects of various thermal properties on the unsteady nanofluid flow that develops a stagnation point flow over a Riga plate. Aspect of the present investigation that can be estimated as follows:

- The Riga plate-subjected nanofluid stagnation point analysis is seen.
- By analyzing implementations of the heat radiation and dissipation phenomenon, the thermal influence of nanofluids is observed.
- Two distinct base fluids-H_2_O and C_2_H_6_O_2_ and Al_2_O_3_ nanostructures develop the nanocomposites.
- A unique model for viscosity and thermal conductivity is utilized.
- The unsteady flow condition is also taken into consideration.
- The simulations for the nonlinear model are computed using the two numerical methods that have shown the most promising, RK-4 and CVFEM schemes.

It has been noted that various researchers have investigated that various nanofluids affect stagnation points flow. The applications of dissipation, thermal radiative phenomena, and unsteady effects for nanofluid (H_2_O/TiO_2_ and C_2_H_6_O_2_/TiO_2_) stagnation point flow have not yet been explored. These objectives are the foundation of this investigation. Through various graphs and tables, the physical impact of flow parameters is taken into consideration. The study suggests implications in recently developed magnetically nano biosensors^38^ and molecular tracking monitoring processes that make use of Riga sensing and magnetic nanocomposites in biochemical manufacturing^39^.

## Physical and mathematical formulation

**Figure 1:**
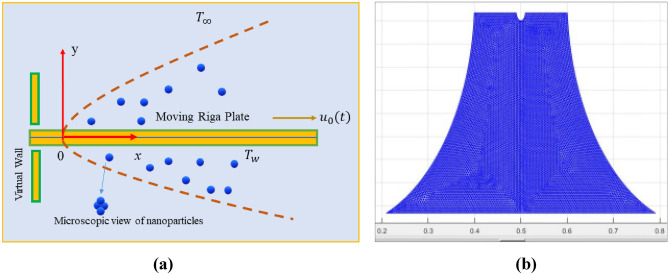
Computational domain of problem with geometry and Grid mesh.

Time dependent, natural convection, incompressible and stagnation state flow of nanofluids H_2_O/TiO_2_ and C_2_H_6_O_2_/TiO_2_ is presented in Fig. 1a are examined subjected to a movable plate under the following some basic assumptions^29,34^:

- $$u_{e} (x,t) = u_{0} (t) + \gamma \left( {x - x_{0} } \right)\left( {t_{rf} - \lambda t} \right)^{ - 1}$$ is the free stream velocity. Here $$u_{0} (t) = \partial x\left( t \right)/\partial t$$ (velocity of moving plate),$$t,t_{rf} ,\gamma ,\lambda$$ are time, reference time, acceleration parameters, unsteadiness parameters with $$\lambda = 0$$(steady case), $$\lambda > 0$$(unsteadiness accelerating case), $$\lambda < 0$$$$\lambda > 0$$(unsteadiness decelerating case)^29,34^.
- In present mathematical model the electro-magnetohydrodynamic effect from the Riga plate is denoted by $\left( {\frac{{\pi j_{0} M_{e} }}{{8\rho_{hnf} }}} \right)e^{{\left( {\frac{{ - \pi y_{1} }}{p}} \right)}}$. Here $M_{e} = \left( {x - x_{0} } \right)M_{0} \left( {t_{rf} - \lambda t} \right)^{ - 2}$, $y_{1} = y\left( {t_{rf} - \beta t} \right)^{ - 0.5}$, $M_{0}$(constant).
- $T_{w} ,\,\,T_{\infty }$ are the wall and free space temperatures.
- Energy expression involves the phenomena of thermal radiation and dissipation.

The preceding formulations are used to model the current problem^29,34^:

1 $$ \frac{\partial \,u}{{\partial \,x}} + \frac{\partial \,v}{{\partial \,y}} = 0, $$

2 $$ \frac{\partial u}{{\partial t}} + u\frac{\partial u}{{\partial x}} + v\frac{\partial u}{{\partial y}} - \frac{{\partial u_{e} }}{\partial t} = u_{e} \frac{{\partial u_{e} }}{\partial x} + \frac{{g\left( {\beta_{T} \rho } \right)_{nf} \left( {T - T_{\infty } } \right)}}{{\rho_{nf} }} + \left( {\frac{{\pi j_{0} M_{0} }}{{8\rho_{nf} }}} \right)\exp \left( {\frac{{ - \pi y_{1} }}{p}} \right) + \upsilon_{nf} \frac{{\partial^{2} u}}{{\partial y^{2} }}, $$

3 $$ \frac{\partial T}{{\partial t}} + u\frac{\partial T}{{\partial x}} + v\frac{\partial T}{{\partial y}} = \frac{{k_{nf} }}{{\left( {\rho c_{p} } \right)_{nf} }}\left( {\frac{{\partial^{2} T}}{{\partial y^{2} }}} \right) - \frac{1}{{\left( {\rho c_{p} } \right)_{nf} }}\left( {\frac{{\partial q^{r} }}{\partial y}} \right) + \frac{{\mu_{nf} }}{{\left( {\rho cp} \right)_{nf} }}\left( {\frac{\partial u}{{\partial y}}} \right)^{2} + \frac{{Q_{h} }}{{\left( {\rho cp} \right)_{nf} }}(T - T_{\infty } ). $$

Here $u$ and $v$ are velocities of nanofluids, $g$(gravitational acceleration) $j_{0}$(current density), $y_{1}$(electrodes), $q^{r}$ is radiative flux. The subscribed *nf* implies simple nanofluid. The Roseland approximations yield the following formulas for radiative flux^27^:

4 $$ q^{r} = - \frac{4}{3}\frac{{\sigma^{*} }}{{k^{*} }}\frac{{\partial T^{4} }}{\partial y}. $$

where $\sigma^{*}$ signify (Stefan-Boltzman constant) and $k^{*}$ indicate (mean absorption coefficient).

Table 1 displays the relationships of the experimentally verified hybrid nanofluid features. A list of the characteristics for H_2_O, C_2_H_6_O_2_, and TiO_2_ for the numerical simulations.

5 $$ \left. \begin{gathered} \chi_{1} = \frac{{\rho_{nf} }}{{\rho_{f} }} = \left( {1 - \phi_{TiO2} } \right) + \phi_{TiO2} \frac{{\rho_{TiO2} }}{{\rho_{f} }}, \hfill \\ \chi_{2} = \frac{{(\rho cp)_{nf} }}{{\left( {\rho cp} \right)_{f} }} = \left( {1 - \phi_{TiO2} } \right) + \phi_{TiO2} \frac{{\left( {\rho cp} \right)_{TiO2} }}{{\left( {\rho cp} \right)_{f} }}, \hfill \\ \chi_{3} = \frac{{\sigma_{nf} }}{{\sigma_{f} }} = \frac{{\left( {1 + 2\phi_{TiO2} } \right)\sigma_{TiO2} + \left( {1 - 2\phi_{TiO2} } \right)\sigma_{f} }}{{\left( {1 - \phi_{TiO2} } \right)\sigma_{TiO2} + \left( {1 + \phi_{TiO2} } \right)\sigma_{f} }}, \hfill \\ \end{gathered} \right\} $$

**Table 1:** Various thermophysical features of base liquids and nanoparticles^28,29^.

Physical features	TiO_2_	H_2_O	C_2_H_6_O_2_
$k\left( {W/mK} \right)$	8.9528	0.613	0.1404
$c_{p} \left( {J/Kg\,K} \right)$	686.2	4179	2048
$\rho \left( {Kg/m^{3} } \right)$	4250	997.1	863
$\sigma \left( {s/m} \right)$	2.6 × 10^6^	5.5 × 10^−6^	0.5 × 10^−6^
$\beta \left( {1/k} \right)$	0.9 × 10^−5^	21 × 10^−5^	57 × 10^−5^
*Pr*	–	6.2	27

Here for $\mu_{nf} \& k_{nf}$ we introduce models^12^ for base fluids C_2_H_6_O_2_ and H_2_O.

## Case 1:

Proposed models for TiO_2_/C_2_H_6_O_2_ nanofluid^12^

6 $$ \to \left\{ \begin{gathered} \frac{{\mu_{nf} }}{{\mu_{f} }} = (306\phi_{TiO2}^{2} - 0.19\phi_{TiO2} + 1), \hfill \\ \frac{{k_{nf} }}{{k_{f} }} = (306\phi_{TiO2}^{2} - 0.19\phi_{TiO2} + 1), \hfill \\ \end{gathered} \right. $$

## Case 2:

Proposed models for TiO_2_/H_2_O nanofluid^12^

7 $$ \to \left\{ \begin{gathered} \frac{{\mu_{nf} }}{{\mu_{f} }} = (123\phi_{TiO2}^{2} - 7.3\phi_{TiO2} + 1), \hfill \\ \frac{{k_{nf} }}{{k_{f} }} = (4.97\phi_{TiO2}^{2} - 2.72\phi_{TiO2} + 1), \hfill \\ \end{gathered} \right. $$

The model problem's flow constraints are^29,34^:

8 $$ \left. \begin{gathered} {\text{At}}\,\,\,y \to 0,\,\,\,u = u_{0} ,v\,\, = \,0,\,\,T - T_{w} = 0,\, \hfill \\ {\text{at}}\,\,\,\,y \to \infty ,\,\,\,u - u_{e} = 0,\,\,\,T - T_{\infty } = 0\,. \hfill \\ \end{gathered} \right\} $$

### Dimensionless analysis

The relevant dimensionless variables for the above model formulation that satisfy equation (1) are^29,34^:

9 $$ \left[ {u,v,\eta ,\Theta ,} \right] = \left[ {u_{0} + \frac{{\gamma \left( {x - x_{0} } \right)}}{{t_{ref} - \lambda t}}f^{\prime}(\eta ), - \alpha \sqrt {\frac{{\upsilon_{f} }}{{t_{ref} - \lambda t}}} f(\eta ),\frac{y}{{\sqrt {\upsilon_{f} \left( {t_{ref} - \lambda t} \right)} }}{, }\frac{{T - T_{\infty } }}{{T_{w} - T_{\infty } }},} \right]. $$

The model equations and boundary conditions of current problem nondimensional version is:

10 $$ f^{\prime\prime\prime} + \chi_{1} \frac{{\mu_{f} }}{{\mu_{nf} }}\gamma \left[ {ff^{\prime\prime} - \left( {f^{\prime}} \right)^{2} + 1 - S\left( {\frac{\eta }{2}f^{\prime\prime} + f^{\prime} - 1} \right)} \right] + \frac{{\mu_{f} }}{{\mu_{nf} }}\chi_{3} MH\exp ( - \Lambda \eta ) = 0, $$

11 $$ \left( {\frac{{k_{nf} }}{{k_{f} }} + \frac{4}{3}Rd} \right)\Theta^{\prime\prime} + \Pr \chi_{2} \left[ {\gamma f\Theta^{\prime} - \frac{S\eta }{2}\Theta^{\prime}} \right] + \frac{{\mu_{f} }}{{\mu_{nf} }}Ec\left( {f^{\prime}} \right)^{2} + \chi_{2} \delta_{h} = 0, $$

12 $$ \left. \begin{gathered} f(0) = 0,\,\,\,\,\,f^{\prime}(0) = c,\,\,\,\,\,f^{\prime}(\infty ) = 1,\, \hfill \\ \Theta (\infty ) = 0,\,\,\,\,\,\,\Theta (0) = 1. \hfill \\ \end{gathered} \right\} $$

In the above equations the prime represents differentiation with respect to $\eta$. The obtained dimensionless parameters are presented as:

$$ {\text{where,}}\,\,\,\left\{ \begin{gathered} Rd = \frac{{4\sigma^{*} T_{\infty }^{3} }}{{k^{*} k_{f} }}\,\,{\text{(Thermal}}\,{\text{radiation}}\,\,{\text{parameter)}}, \hfill \\ \Pr = \frac{\mu Cp}{{k_{f} }}\,\,({\text{Prandtel}}\,\,{\text{number}}), \hfill \\ S = \frac{\beta }{\alpha }\,\,({\text{Unsteadiness}}\,\,{\text{parameter}}), \hfill \\ Ec = \frac{{u_{w}^{2} }}{{\left( {C_{p} } \right)_{f} (T_{w} - T_{\infty } )}}\,\,({\text{Eckert}}\,{\text{number}}), \hfill \\ Gr = \frac{{g\left( {\beta_{T} } \right)\left( {T_{w} - T_{\infty } } \right)x^{3} }}{{\upsilon_{f}^{2} }}\,\,({\text{Grashof}}\,\,{\text{number}}), \hfill \\ \delta_{h} = \frac{{Q_{h} }}{{\left( {\rho C_{p} } \right)_{f} }}({\text{Heat generation parameter}}), \hfill \\ MH = \frac{{\pi j_{0} M_{0} }}{{8\alpha \rho_{f} }}\,\,({\text{EMHD}}\,\,{\text{(Riga}}\,{\text{plate)}}\,{\text{parameter}}), \hfill \\ \Lambda = \frac{{\pi \sqrt {\upsilon_{f} } }}{p}({\text{Width}}\,\,{\text{factor}}\,\,{\text{for}}\,{\text{Electrodes/magnets)}}. \hfill \\ \end{gathered} \right. $$

### Physical quantities

Following are the focused physical quantities, i.e. drag force and rate of heat transfer.

13 $$ \left. \begin{gathered} Cf = \frac{{\tau_{w} }}{{\rho_{nf} u_{e}^{2} }},\,\,\,\,\,Nu_{x} = \frac{{xq_{w} }}{{k_{f} \left( {T_{w} - T_{0} } \right)}},\,\,\,\,\, \hfill \\ \,{\text{where}}\,\,\,\,\,\,\,\,\tau_{w} = \mu_{nf} \left( {\frac{\partial u}{{\partial y}}} \right)_{y = 0} ,q_{w} = - \left[ {k_{nf} + \frac{{16\sigma^{*} T_{\infty }^{3} }}{{3k^{*} }}} \right]\left( {\frac{\partial T}{{\partial y}}} \right)_{y = 0} \hfill \\ \end{gathered} \right\} $$

while the non-dimensional forms result:

14 $$ \left. \begin{gathered} R_{e}^{0.5} Cf = \left( {\frac{{\mu_{nf} }}{{\mu_{f} }}} \right)f^{\prime\prime}(0),\,\, \hfill \\ R_{e}^{ - 0.5} Nu_{x} = - \left( {\frac{{k_{nf} }}{{k_{f} }} + \frac{4}{3}Rd} \right)\Theta^{\prime}\left( 0 \right). \hfill \\ \end{gathered} \right\} $$

## Solution methodology

### Control volume finite element method (CVFEM) procedure

**Figure 2:**
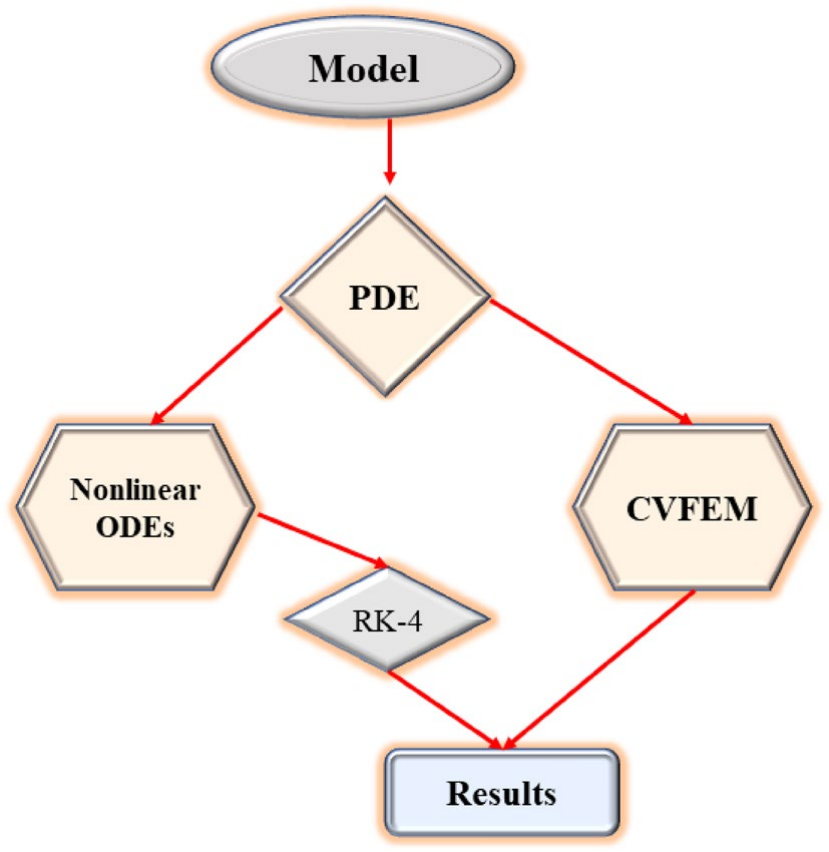
Flowchart of numerical methods^48^.

The Finite element approach, based on the control volume algorithm, is used to computationally carry out the entire simulation, together with the non-dimensional system of equations and their boundary conditions. A recently created method called CVFEM aims to get at a realistic numerical solution to the non-linear system of partial differential equations (PDEs). Sheikholeslami^46,47^ implemented CVFEM for the first time to investigate the heat transmission challenges. The triangle geometry component explores and utilizes multiple physical changes. FEM and FVM are both covered within CVFEM. The use of this strategy in multi-physics challenges with complicated structures is a major benefit. For the appropriate selection of the source and flux, etc. the technique controls the discretization of the geometries from the FEM under the dynamic explanation of the FVM. For the specified geometry, the governing equations are processed to the discretization approach. A system of planetary problems is formed by a mathematical structure that is built up component by component. The Gauss-Seidel scheme is then used to solve the mathematical equation to analyze various state variables and other significant parameters. Figure 1a shows the geometry of the problem and Fig. 1b is the Grids representation obtained from the CVFEM technique. A schematic diagram illustrating the CVFEM methodology is presented in Fig. 2.

### RK (Runge-Kutta) procedure

It is clearly obvious from the fact that transformed differential Eqs. (10), (11) and (12) are extremely non-linear, and it is almost tough to calculate their exact solutions in most situations. To get the approximated solutions to these problems, we generally use a numerical method called the shooting technique with the aid of the fourth order R-K (Runge-Kutta) method^49,50^. By declaring a new set of dependent variables, we convert the existing Eqs. (10), (11) and (12) into a set of first order ODEs, as^49^:

15 $$ \left. \begin{gathered} \xi_{1} = f,\,\,\xi^{\prime}_{1} = \xi_{2} ,\,\,\xi^{\prime}_{2} = \xi_{3} , \hfill \\ \xi^{\prime}_{3} = \chi_{1} \frac{{\mu_{f} }}{{\mu_{nf} }}\gamma \left[ {S\left( {\frac{\eta }{2}\xi_{3} + \xi_{2} - 1} \right) - \xi_{1} \xi_{3} + \left( {\xi_{2} } \right)^{2} - 1} \right] - \frac{{\mu_{f} }}{{\mu_{nf} }}\chi_{3} MH\exp ( - \Lambda \eta ), \hfill \\ \Theta = \xi_{4} ,\,\xi^{\prime}_{4} = \xi_{5} , \hfill \\ \xi^{\prime}_{5} = \frac{{\Pr \chi_{2} \left[ {\frac{S\eta }{2}\xi_{5} - \gamma f\xi_{5} } \right] - \frac{{\mu_{f} }}{{\mu_{nf} }}Ec\left( {\xi_{5} } \right)^{2} + \chi_{2} \delta_{h} }}{{\left( {\frac{{k_{nf} }}{{k_{f} }} + \frac{4}{3}Rd} \right)}}, \hfill \\ \end{gathered} \right\} $$

16 $$ \left. \begin{gathered} \xi_{1} (0) = 0,\,\,\,\,\,\xi^{\prime}_{1} (0) = c,\,\,\,\,\,\xi^{\prime}_{1} (\infty ) = 1,\, \hfill \\ \xi_{4} (\infty ) = 0,\,\,\,\,\,\,\xi_{4} (0) = 1. \hfill \\ \end{gathered} \right\} $$

## Results and discussion

This part comprises the nanofluids flow and energy domain descriptive assessment. To examine the impacts of different model factors on nanofluid temperature and velocity, the entire debate has been depicted through tables and graphs. Except as otherwise stated, we have set the simulation’s parameter values to be $Rd = 0.3,Ec = 10,Gr = 0.3,\,M_{H} = 0.5,\Lambda = 0.1,\phi = 0.01$.

### Impact of physical parameters on velocity distribution

**Figure 3:**
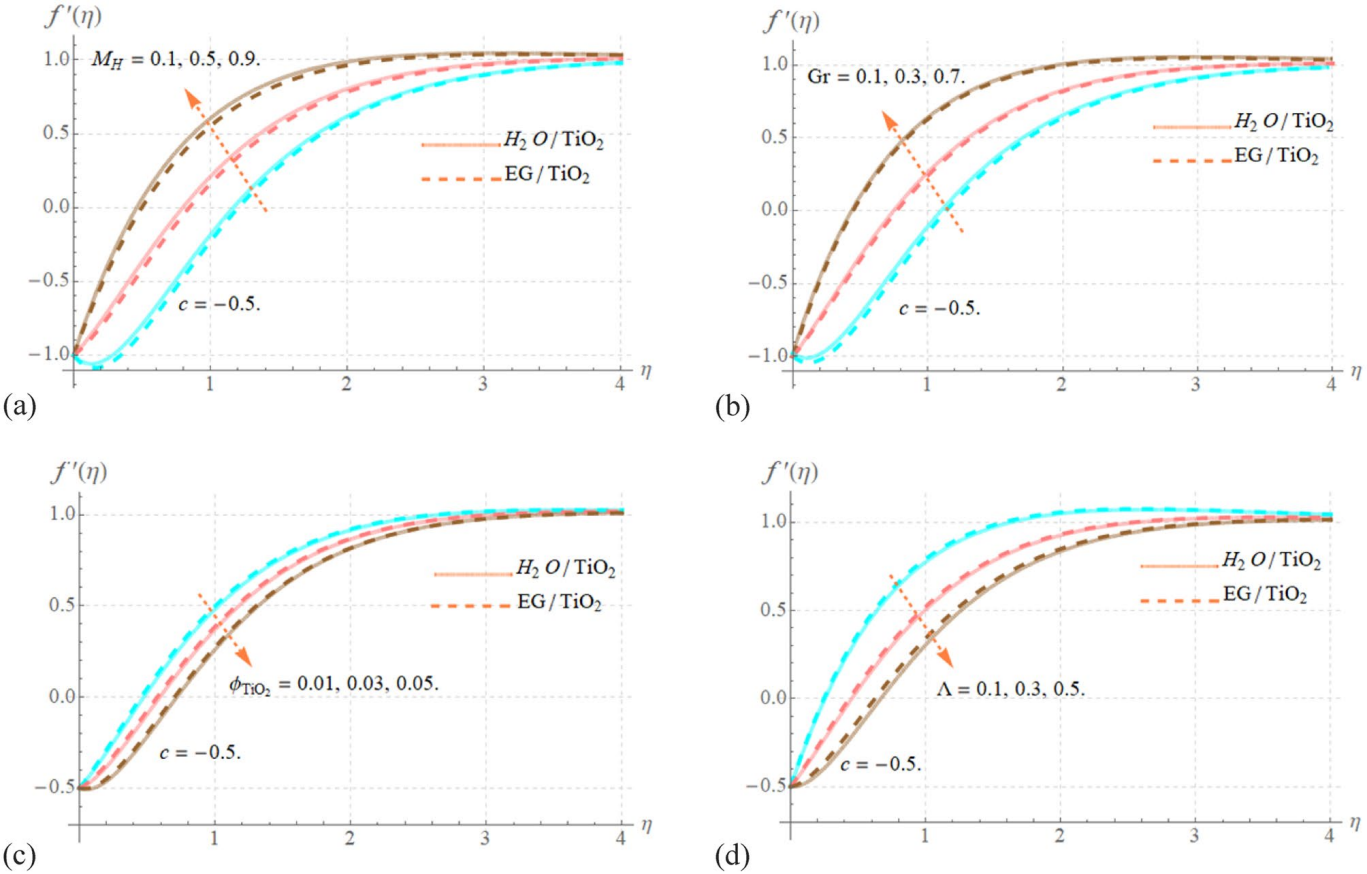
Effect of Gr, M_H_, $\phi_{TiO2}$ and Λ versus $f^{\prime}\left( \eta \right)$.

The plots in Fig. 3a–d explain the various effects of a nanofluids velocity field versus modifications in the magnitude of model factors $Gr,M_{H} ,\Lambda ,\phi_{{TiO_{2} }}$, respectively, while maintaining the value of $c = - 0.5$. The velocity distribution shows an increasing tendency with larger values of $Gr$ and $M_{H}$, as seen in Fig. 3a,b. The Riga concept’s magnets and electrodes are organized in such a way that the resultant Lorentz forces drive the motion of the investigated electrically conducting nanofluid, allowing this analysis to be incredibly reliable. Figure 3b illustrates the variation in the nanofluid velocity field due to the impact of $Gr$.In this scenario, the velocity field grows as the improved $Gr$. Physically, $Gr$ decided how to account for buoyancy in terms of viscous force. As a result, as $Gr$ changed, the velocity field enlarged, and the buoyancy force occurred. The effect of $\phi_{{TiO_{2} }}$ on nanofluids velocity distribution is shown in Fig. 3c. This graph analyzed the findings that the nanofluids speed drops as $\phi_{{TiO_{2} }}$ values rise. This visualizing suggests that increases in boundary layer surface thinning reflect fierce opposition to nanofluid speed. Figure 3d shows the visual findings for the velocity distribution under the variation of $\Lambda$. It shown that when the value of $\Lambda$ provides the maximum fluctuation, the velocity profile exhibits a decreasing behavior. Additionally, it is clear from Fig. 3a,b drawings that though the outputs with increases in $\phi_{{TiO_{2} }}$ are only very little different in both cases, they are significantly more explosive in the case of the C_2_H_6_O_2_ base fluid when compared to H_2_O.

### Impact of physical parameters on temperature distribution

**Figure 4:**
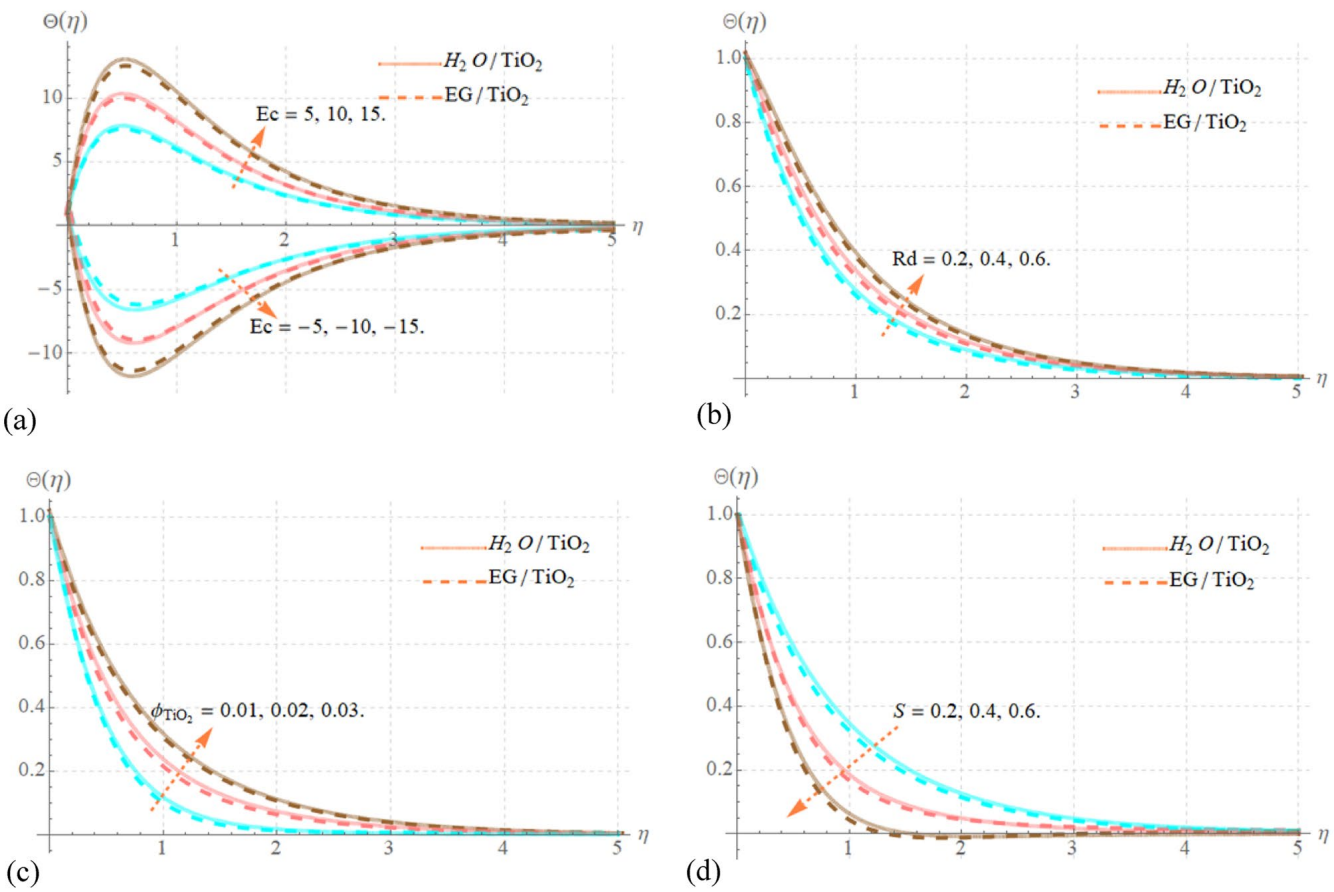
(**a**–**d**) Effect of Ec, Rd, $\phi_{TiO2}$ and S versus Θ(η).

Figure 4a–d are sketched for the nanofluids thermal distribution via $Ec$, $Rd$, $\phi_{TiO2}$ and S parameters. The effect of $Ec$ on dimensionless temperature is clearly shown in Fig. 4a for both increasing and decreasing scenarios. It is revealed that nanofluid thermal profile has significant positive nature for raising values of $Ec > 0$. The Eckert number, from a physical viewpoint, is a measure of the difference between the thermodynamic states of the fluid and the wall, which offers details about the fluid's self-heating properties in high-speed conditions. Frictional energy dissipated as the value of $Ec$ rises because to the viscous interactions between fluid layers, heightening the nanofluid's temperatures. Considering such, the negative attitude is seen to be in contradiction with $Ec < 0$. Figure 4b shows the $Rd$ variability on the thermal profile of nanofluids. As $Rd$ values grow, the temperature profile gets stronger. The influence of higher $Rd$ values on conduction is dominant. The system receives a substantial amount of heat from the radiation, which raises the temperature. Figure 4c demonstrated that the result of $\phi_{TiO2}$ causes an increase in the temperature of nanofluids. The flow field produces thermal energy because of minuscule particles interactions, which raises the fluid's temperature. Hence the injection of $\phi_{TiO2}$ increases the transmission of energy. However, as seen in Fig. 4d, rising levels of $S$ result in a decrease in thermal field. Additionally, like the fluid’s velocity graphs, it is also noticeable from Fig. 4 that the influencing factors show considerable increases when utilizing C_2_H_6_O_2_ instead of H_2_O.

### Exploration of CVFEM findings

**Figure 5:**
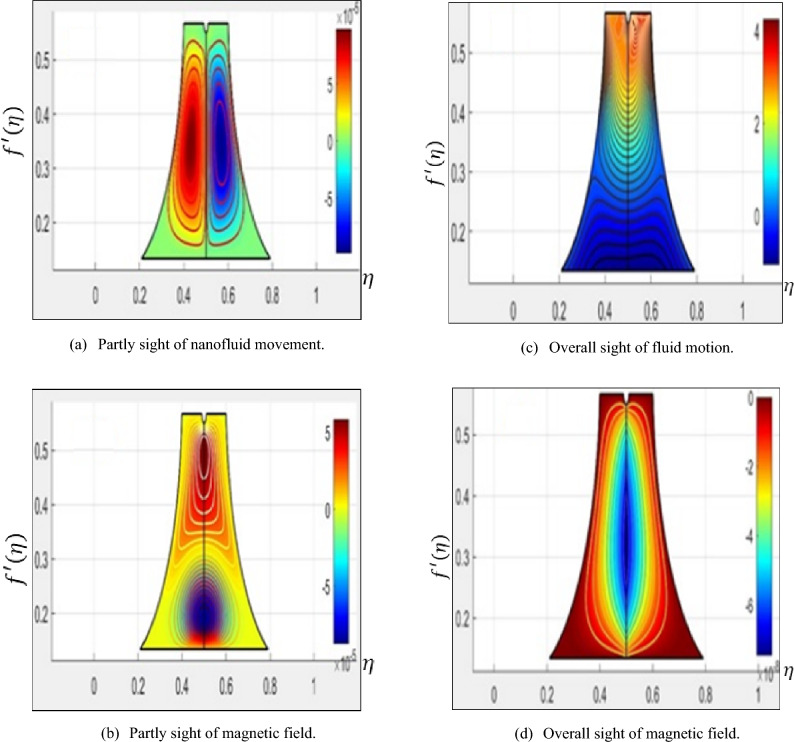
(**a**-**d**) Results of partial and overall analyses of magnetic field and fluid motion from CVFEM.

The illustrations in Fig. 5a–d explain the effects of fluid velocity and the magnetic field from partly and overall viewpoint. Figure 5a–d demonstrate the CVFEM views in portions and as a whole for the aforementioned features, separately. Two scenarios are taken into consideration to invigorate the results. In addition, Fig. 5a and c, which display the velocity contours, and Fig. 5b and d, which simultaneously display the magnetic profiles, respectively.

### Numerical analysis of skin friction and nusselt number

**Figure 6:**
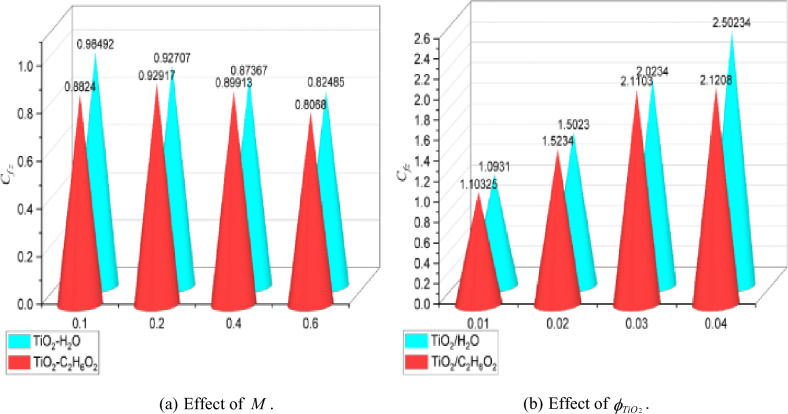
Graphs of ${C}_{f}$ for various values of $M$ and $\phi_{TiO2}$.

**Figure 7:**
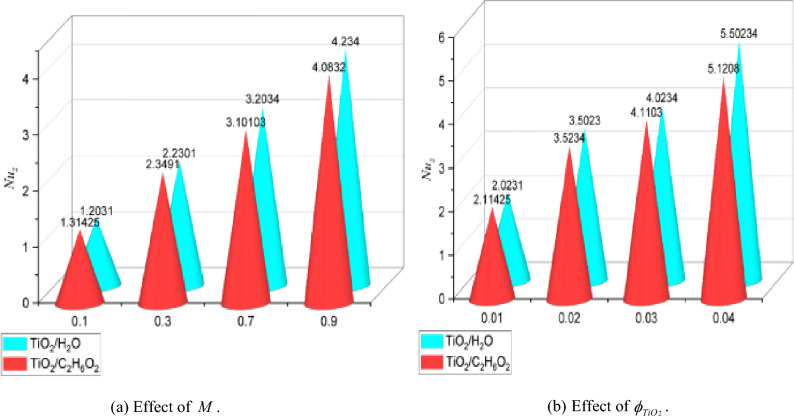
Graphs of $Nu$ for various values of $M$ and $\phi_{TiO2}$.

**Figure 8:**
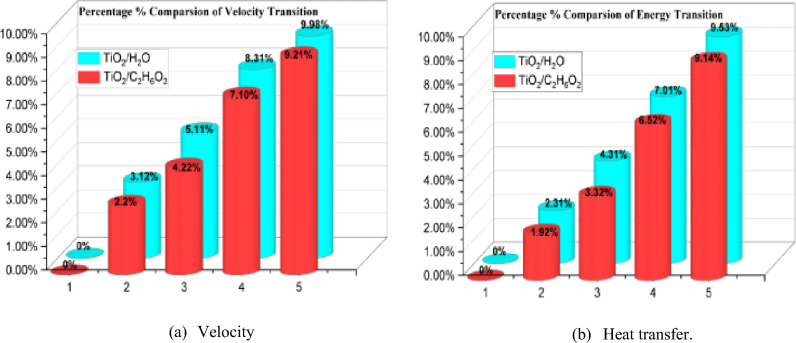
The % comparison between vorticity and energy transmission.

The computational results of skin friction and the Nusselt number in relation to several physical entities were represented graphically in Figs. 6a,b and 7a,b. Moreover, graphs 6 (a, b), both individually, help to explain the effects of skin friction in comparison to $M = 0.1,0.2,0.3$ and $0.6$ and $\phi_{{TiO_{2} }} = 0.01,0.02,0.03$ and $0.04$. In contrast, $\phi_{{TiO_{2} }}$ has adverse effects on *C*_*f*_, which unfavorably affects the flow rate. It is shown that *C*_*f*_ improves against increasing measures of $M$. Figure 7a,b provide a graphic explanation of the effects of $M = 0.1,0.3,0.7,0.9$ and $\phi_{{TiO_{2} }} = 0.01,0.02,0.03$ on the *Nu*. Under rising $M$, *Nu* gradually improves while the beneficial effect against $\phi_{{TiO_{2} }}$ increases. The *Nu*, as seen from the perspective of thermo-fluid mechanics, is the proportion of convectional to conductive heat transmission at a fluid boundary. Therefore, we conclude that, compared to heat conduction, convective thermal transport is much improved for greater values of $\phi_{{TiO_{2} }}$. Additionally, the plots in Figs. 6 and 7 describe a comparison test between the nanofluids made of TiO_2_/H_2_O and TiO_2_/C_2_H_6_O_2_. It should be highlighted that all the results are reported with improvements for H_2_O in comparison to the C_2_H_6_O_2_ base fluid because H_2_O exhibits greater thermal reactions than C_2_H_6_O_2_. Figure 8 shows a percentage-based analysis of heat transfer and vorticity. Nanofluid based on H_2_O performed better than nanofluid based on C_2_H_6_O_2_. Table 2 presents a quantitative evaluation of $f^{\prime\prime}(0)$ versus various acceleration parameter values while keeping the other parameters constant. The assessments from Wang^11^, Ishak et al.^38^, Lok and Pop^39^ and the present are grouped in table. Thus, there is a remarkable agreement of the data. Furthermore, it is noted that $f^{\prime\prime}(0)$ experiences an increase for $\alpha$, although benefits are noticed for positive ranges.

**Table 2:** Validation of $f^{\prime\prime}(0)$ results in previous literature using statistical parameters.

$\alpha$	^30^	^31^	^32^	[Present]
	$f^{\prime\prime}(0)$	$f^{\prime\prime}(0)$	$f^{\prime\prime}(0)$	$f^{\prime\prime}(0)$
− 0.2	0.13278657	0.133573201	0.132762176	0.1336832065
− 1.5	0.321764321	0.3221033254	0.321752183	0.3221033254
− 1.0	1.372183216	1.3733109750	1.372174420	1.37342298342
− 0.5	1.5120265437	1.5131023204	1.5120132321	1.51321321032
0	1.342276489	1.3431230281	1.342175542	1.34323321084
0.5	0.824665432	0.8251202013	0.82451314	0.82522321087
1.0	0	0	0	0
1.5	− 1.215552107	− 1.2141203201	− 1.215424321	− 1.2142134265
2.0	− 1.898533210	− 1.8972312935	− 1.898423214	− 1.8973413287

## Conclusions

In the presence of thermal radiation, the simulation framework for the flow of H_2_O and C_2_H_6_O_2_-based TiO_2_ nanofluids close to the stagnation point pattern with the Riga plate is examined. Analysis of the innovative effects of the current work is delighted with the unsteady relationships with dissipation. CVFEM and the RK-4 scheme are used to computationally solve the model differential equations. To examine the effects of innovative factors on various flow patterns, graphic findings and tabular data are presented. The key points are:

- The velocity profile diminishes when the $\phi_{TiO2}$ parameter is increased, but the fluid velocity is enhanced by the $H_{M}$ parameter.
- Both nanofluids' *C*_*f*_ and *Nu* are improved by the high value $\alpha$. However, for bigger $\alpha$ parameters, H_2_O/TiO_2_ has the higher heat factor and C_2_H_6_O_2_/TiO_2_ has the highest skin friction coefficient.
- By continuously transporting the heat from the fluid particle, $\phi_{TiO2}$ and $Rd$ factors both enhance the thermal profile of both nanofluid.
- Effects of fluid velocity and the magnetic field from CVFEM partly and overall viewpoint in form contours.
- A percentage-based analysis of heat transfer and vorticity are examined and observed that H_2_O based nanofluid performed better than C_2_H_6_O_2_ nanofluid.
- The comparison of TiO_2_ nanoparticles with H_2_O and C_2_H_6_O_2_ base fluid is the only one used in the findings, which are, nevertheless, only compelling. If a different base fluid is utilized, the outcomes can be varied. The thermal development of diverse nanofluids must therefore be investigated in further research. Future studies may want to take the combination of water and ethylene glycol, magnetized hybrid nanofluid, and statistical and numerical data analysis into consideration.

## Data Availability

The datasets used and/or analyzed during the current study are available from the corresponding author on reasonable request.

## References

[1] Bhatti MM, Zeeshan A, Ellahi R, Beg OA, Kadir A (2019). Effects of coagulation on the two-phase peristaltic pumping of magnetized prandtl biofluid through an endoscopic annular geometry containing a porous medium, Chinese. J. Phys..

[2] Turkyilmazoglu M (2022). Multiple exact solutions of free convection flows in saturated porous media with variable heat flux. J. Porous Media.

[3] Turkyilmazoglu M (2022). Exponential nonuniform wall heating of a square cavity and natural convection, Chinese. J. Phys..

[4] Yu W, Xie H (2012). A review on nanofluids: Preparation, stability mechanisms, and applications. J. Nanomater..

[5] Das SK, Choi SUS, Patel HE (2006). Heat transfer in nanofluids—A review. Heat Transf. Eng..

[6] Sidik NAC, Adamu IM, Jamil MM, Kefayati GHR, Mamat R, Najafi G (2016). Recent progress on hybrid nanofluids in heat transfer applications: A comprehensive review. Int. Commun. Heat Mass Transf..

[7] Huminic G, Huminic A (2018). Hybrid nanofluids for heat transfer applications—A state-of-the-art review. Int. J. Heat Mass Transf..

[8] Jamil F, Ali HM (2020). Applications of Hybrid Nanofluids in Different Fields.

[9] Kshirsagar DP, Venkatesh MA (2021). A review on hybrid nanofluids for engineering applications. Mater. Today Proc..

[10] Vallejo JP, Prado JI, Lugo L (2022). Hybrid or mono nanofluids for convective heat transfer applications. A critical review of experimental research. Appl. Therm. Eng..

[11] Suresh S, Venkitaraj KP, Selvakumar P, Chandrasekar M (2012). Effect of Al_2_O_3_–Cu/water hybrid nanofluid in heat transfer. Exp. Therm. Fluid Sci..

[12] Reddy Gorla RS, Sidawi I (1994). Free convection on a vertical stretching surface with suction and blowing. Appl. Sci. Res..

[13] Gul T, Ali B, Alghamdi W, Nasir S, Saeed A, Kumam P, Jawad M (2021). Mixed convection stagnation point flow of the blood based hybrid nanofluid around a rotating sphere. Sci. Rep..

[14] Alnahdi AS, Nasir S, Gul T (2023). Couple stress ternary hybrid nanofluid flow in a contraction channel by means of drug delivery function. Math. Comput. Simul..

[15] Alnahdi AS, Nasir S, Gul T (2023). Ternary Casson hybrid nanofluids in convergent/divergent channel for the application of medication. Therm. Sci..

[16] Hussain SM, Sharma R, Chamkha AJ (2022). Numerical and statistical explorations on the dynamics of water conveying Cu-Al_2_O_3_ hybrid nanofluid flow over an exponentially stretchable sheet with Navier’s partial slip and thermal jump conditions, Chinese. J. Phys..

[17] Bhatti MM, Oztop HF, Ellahi R, Sarris IE, Doranehgard MH (2022). Insight into the investigation of diamond (C) and Silica (SiO_2_) nanoparticles suspended in water-based hybrid nanofluid with application in solar collector. J. Mol. Liq..

[18] Tripathi D, Prakash J, Tiwari AK, Ellahi R (2020). Thermal, microrotation, electromagnetic field and nanoparticle shape effects on Cu-CuO/blood flow in microvascular vessels. Microvasc. Res..

[19] Zeeshan A, Shehzad N, Atif M, Ellahi R, Sait SM (2022). Electromagnetic flow of SWCNT/MWCNT suspensions in two immiscible water-and engine-oil-based newtonian fluids through porous media. Symmetry.

[20] Hussain SM (2023). Numerical assessment of a sutterby hybrid nanofluid over a stretching sheet with a particle shape factor. Waves Random Complex Media.

[21] Hussain SM (2023). Entropy generation and thermal performance of Williamson hybrid nanofluid flow used in solar aircraft application as the main coolant in parabolic trough solar collector. Waves Random Complex Media.

[22] Hussain SM, Jamshed W, Eid MR (2023). Solar-HVAC thermal investigation utilizing (Cu-AA7075/C6H9NaO7) MHD-driven hybrid nanofluid rotating flow via second-order convergent technique: A novel engineering study. Arab. J. Sci. Eng..

[23] Hussain SM, Jamshed W, Pasha AA, Adil M, Akram M (2022). Galerkin finite element solution for electromagnetic radiative impact on viscid Williamson two-phase nanofluid flow via extendable surface. Int. Commun. Heat Mass Transf..

[24] Hussain SM (2022). Irreversibility analysis of time-dependent magnetically driven flow of Sutterby hybrid nanofluid: A thermal mathematical model. Waves Random Complex Media.

[25] Hussain SM (2022). Thermal-enhanced hybrid of copper–zirconium dioxide/ethylene glycol nanofluid flowing in the solar collector of water-pump application. Waves Random Complex Media.

[26] Hussain, S. M., Jamshed, W., Akgül, E. K., & Mohd Nasir, N. A. A. Mechanical improvement in solar aircraft by using tangent hyperbolic single-phase nanofluid. Proc. Instit. Mech. Eng. E J. Process Mech. Eng., 09544089211059377 (2021).

[27] Nasir S, Berrouk AS, Aamir A, Gul T, Ali I (2023). Features of flow and heat transport of MoS_2_+ GO hybrid nanofluid with nonlinear chemical reaction, radiation and energy source around a whirling sphere. Heliyon.

[28] Nasir S, Berrouk AS, Aamir A, Shah Z (2023). Entropy optimization and heat flux analysis of Maxwell nanofluid configurated by an exponentially stretching surface with velocity slip. Sci. Rep..

[29] Dholey S (2021). Unsteady separated stagnation-point flows and heat transfer over a plane surface moving normal to the flow impingement. Int. J. Therm. Sci..

[30] Ishak A, Lok YY, Pop I (2010). Stagnation-point flow over a shrinking sheet in a micropolar fluid. Chem. Eng. Commun..

[31] Lok YY, Pop I (2014). Stretching or shrinking sheet problem for unsteady separated stagnation point flow. Meccanica.

[32] Wang CY (2008). Stagnation flow towards a shrinking sheet. Int. J. Non-Linear Mech..

[33] Ma PKH, Hui WH (1990). Similarity solutions of the two-dimensional unsteady boundary-layer equations. J. Fluid Mech..

[34] Berrouk AS, Lai ACK, Cheung ACT, Wong SL (2010). Experimental measurement and large eddy simulation of expiratory droplet dispersion in a mechanically ventilated enclosure with thermal effects. Build. Environ..

[35] Zainal NA, Nazar R, Naganthran K, Pop I (2022). Magnetic impact on the unsteady separated stagnation-point flow of hybrid nanofluid with viscous dissipation and joule heating. Mathematics.

[36] Gailitis A, Lielausus O (1961). On the possibility to reduce the hydrodynamic resistance of a plate in an electrolyte. Appl. Magnetohydrodyn..

[37] Ganesh NV, Al-Mdallal QM, Al Fahel S, Dadoa S (2019). Riga-plate flow of γ Al_2_O_3_-water/ethylene glycol with effective Prandtl number impacts. Heliyon.

[38] Perez JM, Josephson L, Weissleder R (2004). Use of magnetic nanoparticles as nanosensors to probe for molecular interactions. ChemBioChem.

[39] Supian MZH, Nasir NAAM, Ishak A (2021). Stagnation point flow and heat transfer over an exponentially stretching/shrinking Riga plate with effects of radiation and heat source/sink. Magnetohydrodynamics.

[40] Bilal M, Saeed A, Selim MM, Gul T, Ali I, Kumam P (2021). Comparative numerical analysis of Maxwell's time-dependent thermo-diffusive flow through a stretching cylinder. Case Stud. Therm. Eng..

[41] Ragupathi P, Hakeem AA, Al-Mdallal QM, Ganga B, Saranya S (2019). Non-uniform heat source/sink effects on the three-dimensional flow of Fe_3_O_4_/Al_2_O_3_ nanoparticles with different base fluids past a Riga plate. Case Stud. Therm. Eng..

[42] Ragupathi P, Abdul Hakeem AK, Saranya S, Ganga B (2019). Non-Darcian three-dimensional flow of Fe_3_O_4_/Al_2_O_3_ nanoparticles with H_2_O/NaC_6_H_9_O_7_ base fluids past a Riga plate embedded in a porous medium. Eur. Phys. J. Spec. Top..

[43] Ragupathi P, Saranya S, Hakeem AA, Ganga B (2021). Numerical analysis on the three-dimensional flow and heat transfer of multiple nanofluids past a Riga plate. J. Phys. Conf. Ser..

[44] Hakeem AK, Ragupathi P, Ganga B, Nadeem S (2021). Three-dimensional viscous dissipative flow of nanofluids over a Riga plate. J. Heat Mass Transf. Res..

[45] Abdul Hakeem AK, Ragupathi P, Saranya S, Ganga B (2020). Three dimensional non-linear radiative nanofluid flow over a Riga plate. J. Appl. Comput. Mech..

[46] Sheikholeslami M, Shehzad S, Li Z (2018). Numerical modeling for alumina nanofluid magnetohydrodynamic convective heat transfer in a permeable medium using Darcy law. Int. J. Heat Mass Transf..

[47] Sheikholeslami M, Mahian O (2019). Enhancement of pcm solidification using inorganic nanoparticles and an external magnetic field with application in energy storage systems. J. Clean. Prod..

[48] Wang HF, Anderson MP (1995). Introduction to Groundwater Modeling: Finite Difference and Finite Element Methods.

[49] Khan M, Shahid A, Malik MY, Salahuddin T (2018). Thermal and concentration diffusion in Jeffery nanofluid flow over an inclined stretching sheet: A generalized Fourier's and Fick's perspective. J. Mol. Liq..

[50] Rasheed HU, Khan W, Khan I, Alshammari N, Hamadneh N (2022). Numerical computation of 3D Brownian motion of thin film nanofluid flow of convective heat transfer over a stretchable rotating surface. Sci. Rep..

